# Examining Healthcare Providers’ Knowledge, Attitudes, and Practices in Supporting Pregnant Farmworkers to Mitigate Occupational Pesticide Exposure in California: A Qualitative Study

**DOI:** 10.1177/21501319251407545

**Published:** 2025-12-23

**Authors:** S. Kathleen Steel, Carly Hyland

**Affiliations:** 1 University of California, School of Public Health, Joint Medical Program, Berkeley, USA; 2 University of California, School of Public Health, Division of Environmental Health Sciences, Berkeley, USA

**Keywords:** farmworker health, prenatal care, pregnancy, occupational health, environmental health, occupational pesticide exposure, healthcare providers, healthcare access

## Abstract

**Introduction and Objectives:**

Pesticide exposure during pregnancy is associated with adverse outcomes, and farmworkers face disproportionate risk. In California, healthcare providers can help mitigate exposure through work accommodation notes and State Disability Insurance (SDI) certification. However, little is known about provider knowledge and practices in this area. This study explores how healthcare providers support pregnant farmworking patients in reducing occupational pesticide exposure and identifies key barriers, facilitators, and recommendations for healthcare systems.

**Methods:**

We conducted 3 virtual focus groups and 6 interviews (July 2024-March 2025) with California healthcare providers and state agency staff. Discussions explored knowledge, attitudes, and practices around screening, counseling, and SDI certification for pregnant farmworkers. Transcripts were thematically analyzed using grounded theory with inductive and deductive coding.

**Results:**

Key barriers included limited prenatal care access, inadequate provider training, limited culturally and linguistically appropriate resources, inconsistent screening and counseling practices, and uncertainty around SDI eligibility and certification. These contributed to variable practices: some providers facilitated early disability leave and others hesitated due to unclear guidance or perceived administrative burden. Facilitators included early prenatal care, staff support, knowledgeable providers, and exposure screening workflows.

**Conclusions:**

Standardized guidelines, improved provider education, and coordinated policy and clinic-level changes are urgently needed to ensure equitable care for pregnant farmworkers.

## Introduction

Pesticide exposure during pregnancy is linked to adverse outcomes, including miscarriages, low birth weight, birth defects, and long-term neurobehavioral changes in children.^1-13^ Farmworkers are exposed to higher levels of pesticides compared to the general population, highlighting the importance of targeted interventions to reduce prenatal occupational pesticide exposure in this population.^14-16^ Demographic statistics of farmworkers are variable due to underreporting; however, California is the largest agricultural sector in the United States and employs more farmworkers than any other state.^17^ According to the most recent data from the National Agricultural Workers Survey, 30% of California’s hired farmworkers from 2015 to 2019 were women.^18-21^

Healthcare providers in California are uniquely situated to assist pregnant farmworkers in reducing their occupational pesticide exposure. Two main ways they can do this is by writing work accommodation notes and certifying State Disability Insurance (SDI) claims through the Employment Development Department (EDD). The law requires employers to provide reasonable accommodations to pregnant employees.^22,23^ SDI is entirely employee-funded from payroll reductions and allows eligible workers, regardless of immigration status, to receive income replacement while taking leave from work to avoid harmful exposures during pregnancy.^24^ While pregnancy itself is not considered a disability under the Americans with Disability Act, California law is more expansive and if a pregnant person is exposed to pesticides through their work, they are eligible for paid disability leave for up to 90% of their income for the entirety of their pregnancy.^23-25^

Although healthcare providers can play an important role in supporting farmworking patients, there are no standardized guidelines for the care of pregnant farmworkers, and little is known about how providers address occupational pesticide exposure in this population. Prior studies suggest that clinicians often lack training and confidence in screening and counseling for environmental toxin exposure during pregnancy.^26-31^ For example, in 2014 a national survey of obstetricians revealed that while 78% of respondents believed that environmental health hazards counseling is important during prenatal visits, 50% reported that they rarely take an environmental health history, and less than 20% reported routinely asking about common environmental exposures.^26^ To our knowledge, no recent studies have examined environmental health knowledge and practices among prenatal healthcare providers in California, and none have specifically addressed occupational pesticide exposure.

While resources like factsheets have been developed to support provider education, the uptake of this information remains unclear and research into this topic is scarce.^32,33^ In 2023, interviews with farmworkers in Santa Barbara revealed that physicians were the primary barrier inhibiting patients from accessing disability leave, often telling pregnant patients to continue working even when patients expressed concerns.^21^ Healthcare providers are the gateway for patients to access resources, therefore it is crucial to understand provider knowledge and practices in this area.

This study aims to understand how healthcare providers support pregnant farmworking patients to reduce their occupational pesticide exposure. We conducted focus groups and interviews with pregnancy care providers, as well as employees from the EDD and the Office of Environmental Health Hazard Assessment (OEHHA) in California. We examined providers’ knowledge, attitudes, and practices related to screening and counseling pregnant farmworkers about occupational pesticide exposure, including their involvement in certifying SDI claims. Based on our findings, we identify key barriers and facilitators to supporting these patients and offer recommendations for healthcare systems across California that serve farmworking populations.

## Methods

### Study Design and Setting

We conducted a qualitative study with focus group discussions and interviews. We followed the Consolidated Criteria for Reporting Qualitative Research (COREQ) guidelines to ensure transparency and rigor in study design, data collection, and reporting.^34^ Data collection occurred remotely via Zoom due to participant availability and geographic spread across California. This study was approved by the UC Berkeley Committee for Protection of Human Subjects on May 29^th^, 2024 (Protocol #2024-03-17253). All study participants gave written informed consent.

### Participant Recruitment and Sampling

We used purposive and snowball sampling to recruit focus group participants who provide perinatal care to farmworkers in California. Recruitment was conducted through outreach to clinics serving farmworkers, statewide medical associations, and professional networks. Clinics and associations shared study information with staff and members via email listservs. Interested individuals completed a brief online survey, after which we followed up directly to confirm eligibility, obtain consent, and schedule participation.

Interview participants were recruited either through snowball sampling from focus group participants or directly by the research team due to their relevant professional roles. These roles included either involvement in educational outreach to healthcare providers about pesticides and SDI or experience working in the EDD department that administers SDI.

All participants were aged 18 or older, English-speaking, and either provided perinatal care to farmworkers or were employed by relevant state agencies. All participants provided informed consent, received brief background on the aims of the study, and health care providers received $100 compensation.

### Development of Data Collection Instruments

Focus group and interview guides were developed collaboratively by a multidisciplinary research team, including physicians, legal advocates in farmworker rights, and public health researchers with expertise in environmental and reproductive health. The guides explored: (1) clinical practices around occupational pesticide exposure screening and counseling; (2) provider roles in mitigating pesticide exposure; (3) strategies to improve protection of pregnant farmworkers; and (4) sources of information used to guide clinical practice (Appendix 1). The interview guides were adapted from the focus group guide to include items tailored to each participant’s professional context (Appendix 2).

### Data Collection Procedures

Ninety-minute focus groups and sixty-minute interviews were held via Zoom (version 6.4.12), audio and video-recorded with participant consent, and transcribed using OtterAI (version 3.83.1). Both authors conducted the focus groups and interviews. Transcripts were manually reviewed for accuracy and de-identified prior to analysis. Transcripts were imported into Dedoose (version 10.0.25) for coding and thematic analysis.

### Data Analysis

We employed a combined inductive and deductive thematic analysis approach.^35-37^ An initial codebook was developed based on the interview and focus group guides (deductive) and emergent themes (inductive). Both authors independently reviewed the transcripts and contributed to code refinement. Transcripts were coded by both authors using Dedoose, and discrepancies were resolved through discussion to achieve consensus. We applied grounded theory to identify and synthesize major themes across the focus group and interview data.^34-36^ Themes were organized in tables and illustrated with representative quotes. Both authors reviewed and refined thematic interpretations to ensure analytic rigor and consistency.

## Results

### Participant Demographics

We conducted 3 focus groups with 14 healthcare providers including 6 physicians, 7 nurses, and 1 physician associate in the fields of maternal fetal medicine (MFM), obstetrics and gynecology (Ob-Gyn), family medicine (FM), labor and delivery (L&D), and post-partum care (Table 1). We planned to conduct a fourth focus group with healthcare providers, however, only 1 physician attended and we included it in the study as an interview. After 3 focus groups, we observed strong thematic consistency and no emergence of new themes, suggesting that we had reached thematic saturation. To capture additional perspectives not represented in the focus groups, we conducted 5 semi-structured interviews with a Comprehensive Perinatal Service Program (CPSP) worker, medical assistant, community health worker, and public agency employees at the EDD and OEHHA (Table 2). Individual interviews allowed us to specifically recruit participants whose perspectives were missing from the focus groups and provided scheduling flexibility given healthcare providers’ limited availability. After completing 6 interviews, we again reached thematic saturation and determined that our sample had sufficiently answered our research questions.

**Table 1.** Focus Group Participant Demographics.

Focus group	ID	Title	Specialty	Years of experience	Practice type
1	Participant 1	Physician	Maternal fetal medicine	6-10	Community/teaching
1	Participant 2	Physician	Ob-Gyn	11-15	Community/teaching
1	Participant 3	Perinatal educator, assistant nurse manager	Labor and delivery	21+	Hospital
1	Participant 4	Physician associate	Ob-Gyn	6-10	Private
2	Participant 5	Registered nurse	Labor and delivery	1-5	Inpatient hospital
2	Participant 6	Registered nurse	Post-partum	1-5	Private hospital
2	Participant 7	Registered nurse	Labor and delivery	6-10	Inpatient hospital
2	Participant 8	Physician	Family medicine	16-20	FQHC*, teaching hospital
2	Participant 9	Physician	Ob-Gyn	16-20	Public hospital
3	Participant 10	Registered nurse	Post-partum, labor and delivery, neonatal intensive care	>21	Public hospital
3	Participant 11	Resident physician	Family medicine	1-5	Teaching hospital
3	Participant 12	Registered nurse, public health nurse	Perinatal maternal health	1-5	Public health
3	Participant 13	Physician	Ob-Gyn	>21	FQHC
3	Participant 14	Registered nurse	Labor and delivery	>21	Hospital

* Federally qualified health center.

**Table 2.** Interview Participant Demographics.

Interview	ID	Title	Specialty	Years of experience	Practice type
1	Participant 15	Physician	Ob-Gyn	11-15	Community
2	Participant 16	Comprehensive perinatal services program worker	N/A	>21	Private
3	Participant 17	Medical assistant	N/A	5-10	Public, non-profit
4	Participant 18	Community health worker	N/A	5-10	Public clinic
5	Participant 19	EDD employee	N/A	5-10	N/A
5	Participant 20	EDD employee	N/A	15-20	N/A
6	Participant 21	OEHHA employee	N/A	1-5	N/A

**Figure 1.**
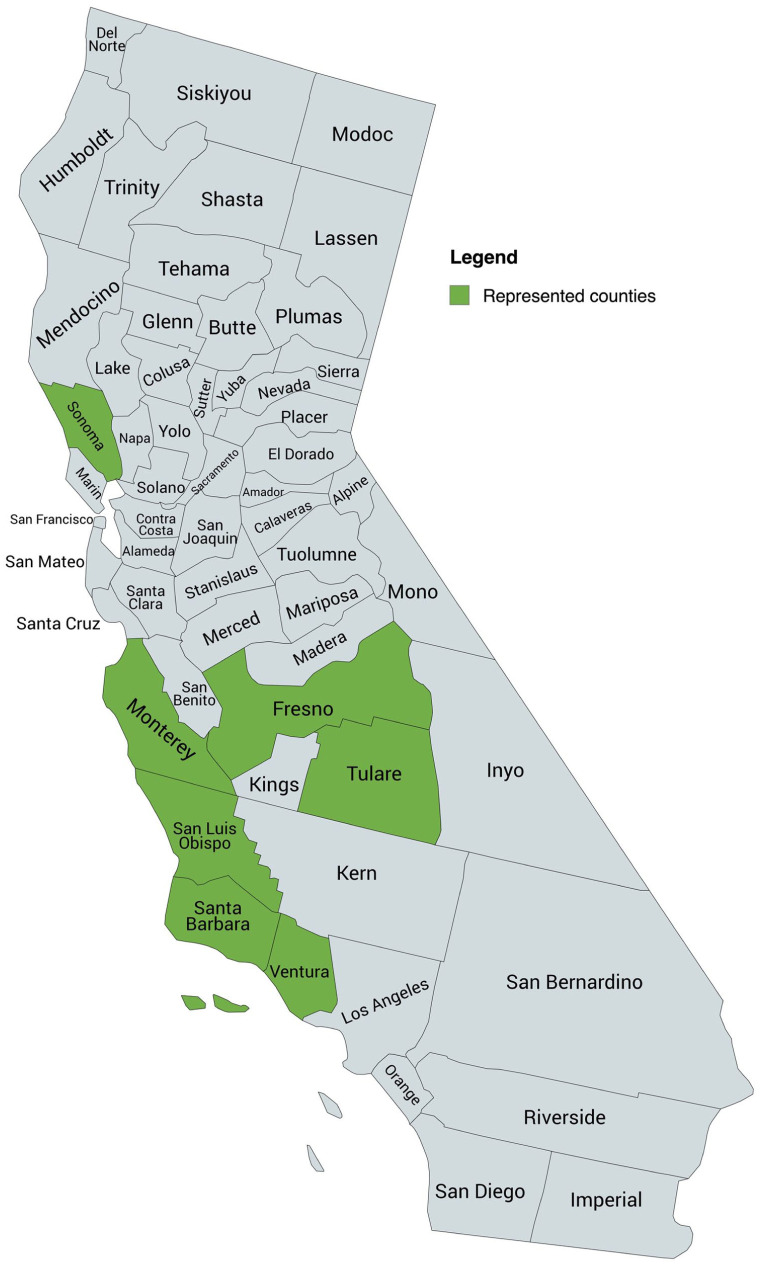
Participant location. Created with mapchart.net.

In total, 21 individuals participated: 14 in focus groups and 7 in individual interviews from July 2024 to March 2025. Eighteen participants (85.7%) were women and 3 were men (14.3%; Table 3). Five participants (23.8%) identified as Asian, 8 participants (38.1%) identified as Mexican, Latino, or Hispanic, and 8 participants (38.1%) identified as white (Table 3). Twelve participants (60%) were 35 to 49 years old, 5 (25%) were 25 to 34 years old, and 3 (15%) were 50 to 64 years old (Table 3). Participants were located in Sonoma, Monterey, Fresno, Tulare, San Luis Obispo, Fresno, Santa Barbara, and Ventura counties (Figure 1).

**Table 3.** Aggregate Focus Group and Interview Participant Demographics.

Characteristic	# (%)
Gender	
Female	18 (85.7%)
Male	3 (14.3%)
Race/ethnicity	
Asian	5 (23.8%)
Mexican/Hispanic/Latino	8 (38.1%)
White	8 (38.1%)
Age*	
25-34	5 (25%)
35-49	12 (60%)
50-64	3 (15%)

* Missing age for 1 participant.

### Thematic Overview

Our analysis identified 5 overarching and interrelated themes that illuminate the structural and interpersonal dynamics influencing prenatal occupational pesticide exposure mitigation: (1) patient barriers to accessing care, (2) factors impacting screening for occupational pesticide exposure, (3) provider roles in counseling and exposure mitigation, (4) barriers and facilitators to certifying SDI claims, and (5) gaps in provider education on environmental health. We examine each theme in detail below. Key facilitators and barriers are summarized in Table 4.

**Table 4.** Summary of Key Barriers and Facilitators to Reducing Occupational Pesticide Exposure During Pregnancy.

Barriers	Facilitators
Patient level • Lack of access to care • Financial burden	Patient level • Access to early prenatal care
Provider level • Lack of provider knowledge about environmental health and options for patients to reduce occupational pesticide exposure • Inconsistent practices across providers	Provider level • Knowledgeable providers
System level • Language barriers, lack of interpreters • Time constraints in clinic • EDD inconsistencies, wait times	System level • Hospital systems that educate providers • Staff support (MAs, CPSP workers) • Language concordance, access to interpreters • Community engagement: ties to the community, more opportunities to interact with medical system

### Barriers to Accessing Care

For healthcare providers to assist patients in reducing their occupational pesticide exposure during pregnancy, patients must be able to appropriately access care. Providers identified access barriers that prevented their patients from receiving early prenatal care, including not being able to miss work for health appointments, concerns about losing their job, financial considerations, and transportation issues. As one physician emphasized “*we’re struggling to get them to engage in the system earlier. I think part of it is just the access*” *- Participant 13 (P13), Ob-Gyn physician.*

Compounding this issue, many hospitals lack appropriate interpreter services for Indigenous languages. One physician described the situation: “*. . .some clinics are definitely well equipped to be able to handle a lot of these social determinants of health and have transportation and interpreters and all of that. We have a lot of clinics that do not have that. And I know that it’s not very equitable”-P1, MFM physician.* Due to this systemic challenge and resource limitation, patients who speak Indigenous languages are not receiving the same quality of care. To navigate these language barriers, it was shared that some patients travel long distances to seek care at clinics that do offer interpreting services (See Supplemental Table 1 for additional supporting quotes).

### Facilitators and Barriers to Prenatal Occupational Pesticide Exposure Screening

Participants in our study had differing practices regarding screening pregnant patients for occupational and environmental exposures. Factors that varied across providers and influenced screening were staff support during intake, standardization of practices, training for providers, the setting of patient encounters, and time limitations. Facilitators to screening for occupational and environmental exposures included incorporating screening questions into clinical workflows and staff support during patient intake. In clinics with these resources, medical assistants (MAs) and Comprehensive Perinatal Service Program (CPSP) workers were crucial to the screening process as they had dedicated time to perform a comprehensive intake.

Barriers to screening included visit time constraints, lack of training and knowledge, and improper setting (eg, postpartum care, specialty care). In clinics with high patient volume and less staff support, environmental and occupational health screening was not prioritized because “*there’s so many things and so little time between patients, because there’s just so many people that need to be seen. I think it makes it hard, and I think a concerted effort would have to be made to add these other questions and discussions [pesticide exposure screening and counseling] on to so many other questions and discussions that have to happen*” - *P13, Ob-Gyn physician.* Several providers indicated that they do not screen due to a lack of training and knowledge about the proper questions to include. For example, a physician shared “*I have an empiric idea of how to do it [screen], but to be honest, we don’t have a special training for this. . . I would say that the main reason [I do not screen] is maybe because of the lack of training*” - *P15, Ob-Gyn physician* (See Supplemental Table 2 and 3 for additional supporting quotes).

### Provider Involvement in Supporting Patients: Factors Impacting Occupational Pesticide Exposure Mitigation Counseling During Pregnancy

Healthcare providers supported pregnant farmworking patients to reduce pesticide exposure through individual counseling about risk mitigation (eg, wearing personal protective equipment (PPE), washing hands and clothes after working in the fields), work accommodation notes, and the certification of SDI claims. Several physicians highlighted that taking disability is the primary and most effective method chosen by their patients to reduce pesticide exposure during pregnancy. Other providers began by counseling about potential work modifications and wrote accommodation notes before certifying disability claims. Productive patient-provider conversations surrounding pesticide exposure employed shared-decision making and acknowledged the tensions facing their patients, including financial considerations, job security concerns, and realistic risk mitigation strategies given each patient’s unique situation.

Barriers to patient counseling included inappropriate setting (eg, labor and delivery, postpartum care), insufficient time, lack of training and education, and mistrust. Some participants brought up patient-provider power dynamics and cultural factors that influenced counseling. For example, providers were aware that their patients may not always voice their concerns or be comfortable speaking up against provider recommendations. A CPSP worker observed: “*also the way our community expresses themselves to what their needs are, they don’t say anything. You have to seek the information*” - *P16.* A physician reflected that their *“. . .farmworker patients tend not to debate a lot. . . I always feel sensitive to the fact that they’re not necessarily going to tell me how they feel about counseling that I give them*” - *P9, Ob-Gyn physician.*

Providers discussed the balance of ensuring their patients are informed about the risks of pesticide exposure while also not wanting to make patients feel guilty if they are unable to make modifications. A physician summarized: “*to be honest, it is really hard to have the conversation sometimes with patients about when they are working in the fields, because they have so little control. . .and then you don’t want to make them feel bad if they do decide to work . . . it’s kind of a balance. . .These are the options that you have, but doing it in a way that doesn’t make them feel guilty, like have that mom guilt, you know, but also empowering them with the information*” - *P9, Ob-Gyn physician.*

Financial considerations were a primary factor in counseling discussions and providers were aware of and sensitive to the pressures facing their farmworking patients. One physician noted “*I don’t want to assume that when I’m taking them off work that everything’s going to be fine at home. If they can’t financially afford to be off work, we kind of talked about the risks and benefits of being off work* versus *not*.” - *P1, MFM physician.* As with environmental exposure screening, counseling discussions were difficult to have in busy, time-pressed clinical settings because “. . .*you’re literally scheduled for just a few minutes to get heart tones and an ultrasound. It’s not set up in a way that really leads to a lot of that anticipatory guidance, and any sort of environmental health, always ends up falling lower down in the priority list because they’re having some bleeding or they’re nauseous, you know, first trimester, there’s so many other things to address*” - *P9, Ob-Gyn physician* (See Supplemental Table 4 for additional supporting quotes).

Additionally, for patients who decided to continue working during pregnancy, there were issues outside of the medical system that impaired their ability to reduce occupational pesticide exposure. Providers reported hearing from patients that employers failed to adequately protect their workers by not providing PPE and spraying pesticides while workers were nearby.

### Barriers and Facilitators to Certifying State Disability Insurance Claims

There were variable attitudes, knowledge, and practices among the providers in our study regarding the use of occupational pesticide-related disability certifications for pregnant patients. Broadly, there was a lack of provider knowledge about the eligibility criteria and use of occupational pesticide-related disability for pregnant farmworkers. Concerns about fraud, “working the system,” and overwhelming the EDD office with claims led some providers to not offer occupational pesticide-related disability to their pregnant patients. One physician revealed: “*I talked to the folks [in a nearby county], they say that there’s a lot of doctors out there who are really reluctant, they feel like they’re committing fraud if they say that their patients should be out of work for pesticide exposure because they’re not actually disabled.*” - *P9, Ob-Gyn physician* and another physician expressed concerns: “*If we started giving disability to everyone who asked for it all at once, it would be a deluge of paperwork for the office and a lot of expense for the disability system*” - *P13, Ob-Gyn physician.*

Additionally, most hospitals and clinics did not offer clear guidelines to providers about SDI certification. Instead, providers learned through word of mouth and often one provider took the initiative to educate themselves and their colleagues on their patient’s rights. The way providers used disability certification varied. Some providers offered occupational pesticide-related disability to their patients as soon as the first positive pregnancy test or their first visit. Other providers certified disability claims later on in pregnancy in the second and third trimesters.

Facilitators to a successful SDI claim submission and approval process were staff support, a clinical culture that supported SDI certification and educated their providers on patient eligibility, and forms being filled out completely and correctly. MAs and CPSP workers were the primary staff who filled out and submitted disability forms. One provider noted that patients who had access to CPSP services were more educated in general about their disability options.

Providers discussed several barriers that they and their patients faced when accessing occupational pesticide-related disability during pregnancy. There was insufficient time in clinic to fill out forms and communicate with the EDD if more information was needed for claim approval. Providers found it difficult to contact the EDD office and experienced unresponsiveness (eg, calling the phone line with no answer) if they had inquiries about a claim. Providers reflected that many patients were concerned about financial stressors and potential income loss that could result from stopping work and therefore were hesitant to go out on disability. Examples of patient concerns included no guarantee that the claim would be approved, SDI only supplementing a portion of their income, and long wait times to receive payment. Additionally, some providers experienced denials with first trimester pesticide-related claims using the ICD-10 code Z57.4 “Occupational exposure to toxic agents in agriculture” and inconsistencies with claim approval. To combat these claim denials, providers made arguments to the EDD on why this claim should be approved and submitted additional ICD-10 codes (eg, M54.5 lower back pain, 026.892 abdominal pain affecting pregnancy [other specified pregnancy related conditions, second trimester], and R10.2 round ligament pain [pelvic and perineal pain]; See Supplemental Table 5 for additional supporting quotes).

### Environmental Health and Pesticide Education

Overall, there was a lack of provider education surrounding environmental health and ways that providers can help patients mitigate occupational pesticide exposure during pregnancy. Some providers attributed this deficit to gaps in curriculum and training in their schooling. Additionally, pesticide mitigation options were not included in any onboarding training for the providers who participated in our study, despite farmworkers constituting a majority of their patient population. Furthermore, a physician noted that there has been a lack of information on this topic distributed by trusted medical organizations, stating that “*I wouldn’t necessarily say ACOG has provided a ton of information, you know, it’s really all of us. . .that are doing this stuff day in and day out, where we kind of figure out what is best for our patients*” - *P1 MFM physician.*

As such, individual providers have learned on the job and taken on the responsibility to educate themselves and their colleagues. For example, a resident physician shared: “*I haven’t had any formal education, but mine is specifically experience and working with the providers that I do and seeing how they practice, and them helping me inform my practice as a budding clinician*” - *P11, FM physician.*

Providers with proximity to pesticide-related health effects research certified disability claims for their patients at the earliest opportunity and encouraged their colleagues to do the same. A physician explained: “*I did my residency down in [a farmworking county], and we had a lot of farm workers too, but I didn’t really know about the effects of pesticides until I started working up here in Salinas. And really it didn’t really dawn on me until our organization participated in the CHAMACOS study and since then, basically brought awareness to that and so I try to be an advocate for it [early disability certification]*” - *Ob-Gyn physician.*

### Solutions

Providers identified strategies to reduce occupational pesticide exposure, including improving access to care so patients can seek help earlier and use resources like SDI to take leave from work as soon as they learn they are pregnant. Providers recognized that to enable this, patients need to be educated on their rights and options during pregnancy in a format that is effective and both culturally and linguistically concordant. Providers noted that many patients will continue to keep working during pregnancy and that educational materials should include ways to mitigate pesticide exposure risk while working (eg, proper PPE use). Some providers suggested that short videos or pamphlets would be an effective way to convey this information to patients. Providers mentioned the importance of community outreach to distribute information and build trust. Several participants shared experiences trying to organize community outreach events but faced obstacles due to lack of time and resources.

Education for providers was another solution that was frequently discussed. A physician explained why they lean into education: “I don’t know if, well, I’m talking about policies and politics. I’m pretty sure we can do something there, but I’m not an expert on those topics. I believe in education. I believe in the everyday work. So, I think that will be a really good start to educate ourselves and educate our patients. . .because to be honest, we lack that education in our clinic” - P15 Ob-Gyn physician. Providers emphasized the importance of environmental health education, including pesticide-related screening and counseling training, and education for providers about the rights and options of their patients (eg, disability eligibility). Providers expressed the desire for this training earlier on in their careers, for example a family medicine resident shared “I didn’t learn about it at all in medical school, and only now in residency because of the patient population that we work with, I think, is it such an important thing that we communicate and learn about in residency. So genuinely education, especially about the patient population that you’re taking care of” - P11, FM physician. An OEHHA employee involved in provider education affirmed this sentiment: “starting that education and training earlier in young physicians’ careers, so that it just becomes more commonplace, and not something that’s a shock to hear way down the line” - P21. Challenges that providers have faced in education included provider turnover, certain providers not being willing to change their practices, limited time to receive education, and requirements being difficult to standardize.

Suggested formats for delivering this education included integrating the topic into medical school curricula, requiring continuing education courses, and using short-form videos. The EDD outreach office offers webinar trainings for providers about how to navigate the SDI certification process, however, the EDD cautioned that they must be “*very careful on telling them [physicians] how to do their job. In fact, we’re directed not to tell them how to do their job. They are the ones providing their medical expertise, and we as the department have to go by what they are certifying. . .But if they’re believing that this is not proper then we’re stuck, you know, that’s it, right? And that’s how far EDD can go*” - *P19*, meaning that while the EDD can offer education on the eligibility requirements for pesticide-related SDI and support in navigating the claim process, they cannot provide medical expertise on when to offer patients disability.

Hospital-based solutions suggested by providers included improved screening procedures for occupational pesticide exposure, better mechanisms for connecting patients to resources, patient education materials linked directly into the electronic medical record for discharge instructions, and utilizing community health or CPSP workers. Providers indicated a need for improvements to the EDD website to make instructions and eligibility requirements for occupational-pesticide related SDI more clear and additional training for EDD staff to increase consistency in claim approval. Advocacy and policy change were solutions that came up less frequently compared to patient and provider education. However, a few providers suggested advocating for reimbursement for filling out paperwork and speaking out against employers with harmful practices as ways to contribute to reducing occupational pesticide exposure for pregnant patients.

## Discussion

Through focus groups and interviews, we examined how California perinatal healthcare providers screen, counsel, and support pregnant farmworkers in reducing occupational pesticide exposure, including the use of SDI to facilitate work leave during pregnancy. Conversations with providers and state agency staff revealed prenatal care access barriers for patients, gaps in provider training, limited culturally and linguistically appropriate resources, inconsistent screening and counseling practices, and uncertainty around SDI eligibility and certification. These barriers contributed to highly variable provider approaches, with some facilitating early disability leave and others hesitant to certify SDI claims due to unclear guidance or perceived administrative burden. Facilitators for helping pregnant patients mitigate occupational pesticide exposure included early access to culturally and linguistically appropriate prenatal care and support from CPSP staff and MAs. Provider knowledge about pesticide-related health effects and patients’ options to reduce pesticide exposure during pregnancy also played a key role. Additional facilitators were the integration of pesticide exposure screening and counseling into clinic workflows, as well as clinic environments that were actively supportive and engaged in assisting patients to reduce their occupational pesticide exposure.

### Patient Barriers to Accessing Care

Providers in our study discussed high costs, lack of interpreters, transportation issues, and patient concerns about missed wages and losing their job as barriers that prevented patients from seeking prenatal care. It is California law for patients to have access to qualified interpreting services, yet language access is a documented issue, particularly for patients who speak Indigenous languages.^38-40^ Previous studies have supported our findings and additionally found that healthcare access is limited in farmworking populations due to lack of insurance and fear of deportation.^41-43^

Most of the data collection for our study took place before the 2024 election. We hypothesize that themes such as fear of deportation and concerns about public charge may have emerged more prominently had the focus groups and interviews been conducted after January 2025. Access to care barriers in farmworker populations will continue to be exacerbated by current federal government policies as many people will delay seeking healthcare out of fear.^44^ This study adds to the growing body of literature examining how our current healthcare system can fail to meet the needs of patients and underscores the importance of systemic change to better support and care for marginalized populations.

### Provider Education Gaps Lead to Variable Practices

We found that across all levels of care and various locations in California, there was a lack of education and standardized practices for helping pregnant patients reduce their occupational pesticide exposure. Environmental health education is not prioritized in medical training, which was reflected in the knowledge, attitudes, and practices of the providers in our study. While previous studies and organizations have called for the incorporation of environmental and occupational health topics into medical training, our findings indicate that gaps in provider knowledge about these topics persist.^28,45^ We found that participants reported receiving no pesticide-specific environmental health training during their medical education or job onboarding, despite primarily serving farmworking communities. As a result, providers demonstrated inconsistent approaches to screening, counseling, and certifying patients for SDI. For example, providers certified disability claims at different gestational ages, sometimes even within the same clinic system.

Many providers expressed concern about prenatal occupational pesticide exposure and a strong desire for more training and clear guidance. Several even joined the focus groups specifically to learn more and apply this knowledge in their clinical practice. While some providers had previously taken the initiative to educate themselves and often became catalysts for change within their clinics and by sharing knowledge with colleagues, these efforts were largely individual rather than system-driven. The educational gaps are compounded by the lack of standardized clinical guidance from trusted medical organizations such as ACOG, which has not released specific prenatal care recommendations for patients exposed to occupational pesticides. Without clear guidelines or established clinic protocols, the responsibility for addressing this complex issue falls disproportionately on individual providers rather than being supported through coordinated, top-down approaches.

### Environmental Health is Deprioritized

Deprioritization of environmental health was a common theme we encountered. Due to the structure of our current healthcare system, providers were overbooked and had minimal time for prenatal visits which resulted in urgent medical concerns taking priority over pesticide screening and counseling. Furthermore, environmental and occupational health screening and counseling was not officially integrated into the clinic workflows for any of our participants, placing the burden on providers to initiate those conversations during the visit. Additionally, providers described that some patients chose to continue working in the fields throughout pregnancy or delayed seeking care due to concerns about missed wages and losing their job. While addressing prenatal occupational pesticide exposure in a clinical setting is challenging, there are several steps clinics and providers can take to better support their patients.

For example, we found that staff support during intake and incorporating questions about occupation and environmental exposures into clinical workflows was crucial to ensuring that prenatal pesticide exposure was being captured and addressed. MAs and CPSP workers were particularly important and played a key role in screening for occupational pesticide exposure and helping patients navigate the SDI claim process. This supports the necessity of these positions in a clinical setting, especially for marginalized populations experiencing barriers accessing healthcare.

### State Disability Insurance

In a 2021 survey of agricultural workers in California, only 37% of participants were told by their doctor to stop working at any point in their pregnancy.^43^ This percentage varied greatly across different counties in California: 45%-58.3% in the Central Coast, 34.3% in San Joaquin Valley, 32.8% in Napa and Sonoma, and just 27% in Imperial and Coachella Valley.^43^ This further demonstrates that there is a lack of widespread physician knowledge about how to support pregnant patients exposed to occupational pesticides, likely due to an absence of standardized guidelines across California. In the same study, it was found that among doctors who did recommend leave, the median gestational age was 16 weeks.^43^ This variable timing aligns with findings from our study. We found several factors that impacted the gestational age at which patients were offered disability for their occupational pesticide exposure. First, access to care barriers resulted in many patients attending their first prenatal visit at a later gestational age. Additional factors were the lack of standardized clinic guidelines regarding SDI certification for this patient population and a lack of provider knowledge about SDI eligibility, including when disability claims can be certified for occupational pesticide exposure. While providers acknowledged the health risks associated with pesticide exposure during pregnancy and agreed that patients should avoid pesticide exposure at the earliest opportunity, some providers had concerns about the legitimacy of certifying pesticide claims early in pregnancy and were more accustomed to offering disability later for other medical complaints, such as back pain. Conversely, providers in our study who were knowledgeable about the health effects of pesticides and the rights of their patients, certified occupational pesticide-related disability claims for their pregnant patients at the earliest opportunity. This aligns with recommendations from groups such as OEHHA and Pregnant@Work that advise providers to offer disability leave to pregnant farmworking patients at the first positive pregnancy test.^32,46^ In our interview with EDD employees, they told us that patients who are exposed to occupational pesticides are eligible for disability as soon as they know they are pregnant. The EDD made it clear that if the claim is filled out correctly and the certifying physician explains why the patient needs to come off work with the appropriate ICD-10 codes, the claim will be approved, provided other eligibility criteria are met (eg, earned at least $300 with SDI deducted from their paycheck).

Despite assurance from the EDD that all correctly completed occupational pesticide-related claims would be approved, MAs and CPSP workers in our study described occasionally getting denials for claims submitted at an early gestational age. To get these claims approved, the clinic staff had to go back and add additional ICD-10 codes and justification for the disability leave. A delay in claim approval puts financial pressure on patients as they must wait longer for income replacement. Additionally, providers described that it was difficult to contact the EDD to clarify claims, further prolonging the approval process. Providers in our study believed that inconsistencies in claim approvals stemmed from a lack of understanding within the EDD regarding eligibility criteria and the approval process.

### Structural Problems, Individualized Solutions

We found that providers recognized and named structural and system-level failures that impacted how they screen, counsel, and support patients in reducing their occupational pesticide exposure. These included visit time constraints, patients’ lack of healthcare access, interpreter deficits, high costs, and gaps in standardized clinic practices. However, when asked about solutions, providers overwhelmingly named an individual level solution– education. As one provider explained, this tendency may stem from the healthcare community’s familiarity with education and training as areas where they feel a sense of agency and control. In contrast, structural challenges, such as those requiring policy changes or large-scale hospital system reforms, may feel unachievable as an individual. As a result, providers often default to interventions within their immediate reach, ultimately assuming even more responsibilities in a system that already leaves them overburdened. Additional burden is also placed on the patients, as the onus is on them to advocate for themselves in clinical settings. Our findings highlight a central tension: although providers recognize pesticide exposure as a structural and systemic issue affecting pregnant farmworkers, many of the solutions currently offered at the clinical level remain individualized and rely heavily on patient and provider action.

While education for both providers and patients is needed and should be developed in collaboration with the community it aims to serve, systemic change is also required to best support patients. Potential opportunities for policy-level action include establishing state-level training requirements for perinatal providers on pesticide exposure, improving consistency and clarity across EDD protocols, and expanding support for Indigenous-language interpretation services to ensure equitable access to healthcare. These systemic changes could enhance providers’ ability to offer comprehensive and linguistically concordant care to pregnant farmworking patients.

### Strengths, Limitations, and Future Directions

Due to the qualitative design of our study, a strength was the richness of data that we gathered through open-ended focus group discussions and interviews. This allowed themes to arise and gave providers an opportunity to discuss topics and issues that they felt were important. The focus groups created a space for providers to come together to brainstorm and learn from each other and served as an opportunity for community and engagement in work to improve patient care. Many providers commented how they learned a lot from the sessions and were going to start incorporating screening and counseling for occupational pesticide exposure into their clinical practice as well as educate their colleagues on the topic. This demonstrated that there is a desire and need for education in this area.

Our study had several limitations. It is a small sample size and not representative of all pregnancy providers in California. The sample is geographically concentrated in the Central Coast region of California which may limit the transferability of findings to other agricultural areas with different workforce characteristics, clinical infrastructures, or regulatory environments. Additionally, participants who elected to take part in the study may have had greater prior interest in the issue of pesticide exposure during pregnancy and therefore may have known more about the topic and been more willing to support farmworking patients. This potentially introduced a selection bias that influenced our results. Due to these factors, we hypothesize that pregnancy providers in California may be less knowledgeable about how to address pesticide exposure during pregnancy and have more varied practices than we report in our findings. Furthermore, data collection was conducted during a specific sociopolitical period, and evolving state and federal policies and current events may have influenced how providers approached pesticide exposure during pregnancy. These limitations should be considered when interpreting and applying the study’s findings.

Future studies should aim to recruit a broader range of providers across California and nationally, with efforts to include those who are not already advocates for farmworking populations. This will ensure a more comprehensive representation of perspectives. Additionally, future research should incorporate questions addressing documentation status and fear of seeking care as potential barriers to care access as these were factors we did not fully assess due to the timing of our study.

## Recommendations

Through incorporating the information, we gathered from our study and existing literature, we have compiled recommendations for hospitals and providers to better care for pregnant patients exposed to occupational pesticides.

- Incorporate environmental health education into early medical training (eg, medical school, residency)
- Require onboarding training for providers about their farmworking patient population that includes how to screen, counsel, and support pregnant patients in reducing occupational pesticide exposure
  - Include training on SDI eligibility and certification process
- Increase staffing, especially MAs and CPSP workers, to support pesticide screening, counseling, and disability certifications
- Standardize clinical care workflows and guidelines to include the following:
  - Screening for occupational pesticide exposure
  - Counseling patients about the risks of pesticide exposure
  - Counseling about options patients have to reduce their occupational pesticide exposure during pregnancy
- Guidance to certify pesticide related disability claims using ICD-10 code Z57.4 “Occupational exposure to toxic agents in agriculture” as early as the first positive pregnancy test or as soon as their first clinic visit
- Address access to care barriers and the urgent need for Indigenous language interpreters
- Advocate for policy changes that supports this patient population
- Urge ACOG and other medical organizations to release farmworker-specific care guidelines that include the health implications of pesticide exposure during pregnancy on the pregnant person, pregnancy, and future child

These recommendations may require different implementation approaches depending on clinic structure and resources. For example, Federally Qualified Health Centers (FQHCs) may more easily integrate pesticide exposure screening into existing CPSP workflows. Under resourced clinics with limited staffing may benefit from integrating occupational exposure questions directly into the electronic medical record. In larger hospital systems it may be possible to incorporate environmental health training into existing grand rounds or required continuing education.

## Conclusion

Healthcare providers can play a crucial role in assisting pregnant patients to reduce their occupational pesticide exposure through screening, counseling, and connecting patients to resources. However, provider efforts are constrained by systemic factors such as the depriotization of environmental health, lack of interpreter services, time pressures, and inadequate institutional guidance. To meaningfully and sustainably reduce prenatal occupational pesticide exposure, interventions must extend beyond individual-level education to include systemic and structural change. Our findings highlight a need for coordinated policy and clinic-level reforms that establish standardized care guidelines, integrate environmental health into provider training, and expand prenatal care access to ensure consistent and equitable support for pregnant farmworkers.

## Resources

Below are resources and clinical guidelines that are available for providers to become better educated on these topics.

- Essential Information for Healthcare Providers: Pregnant Agricultural Workers and Pesticides: https://oehha.ca.gov/sites/default/files/media/downloads/pesticides/general-info/pregnancypesticidesinfosheet.pdf
- Dar a Luz: Legal Rights for Farmworkers in Pregnancy and Postpartum: https://worklifelaw.org/dar-a-luz/
- Guidance for Medical Care Providers: Supporting Farmworkers during Pregnancy and Postpartum: https://pregnantatwork.org/guidance-medical-provider-agriculture/
- California State Disability Insurance for Farmworkers During Pregnancy: https://pregnantatwork.org/wp-content/uploads/SDI-For-Farmworkers-During-Pregnancy.pdf

## Supplemental Material

sj-docx-1-jpc-10.1177_21501319251407545 – Supplemental material for Examining Healthcare Providers’ Knowledge, Attitudes, and Practices in Supporting Pregnant Farmworkers to Mitigate Occupational Pesticide Exposure in California: A Qualitative Study Supplemental material, sj-docx-1-jpc-10.1177_21501319251407545 for Examining Healthcare Providers’ Knowledge, Attitudes, and Practices in Supporting Pregnant Farmworkers to Mitigate Occupational Pesticide Exposure in California: A Qualitative Study by S. Kathleen Steel and Carly Hyland in Journal of Primary Care & Community Health

## Appendix

### 1: Focus Group Guide

1. Ice breaker: Can we go around the room and, in 1 min, share your first name, professional title or role, and 1 thing you love about being a pregnancy healthcare provider?
2. Transition question: What proportion of your pregnant patients are farmworkers?
3. Content questions
  - Content #1- Comfort and responsibility on counseling for pesticide exposure: Do you screen and counsel your pregnant farmworker patients on the health effects of pesticide exposure?  
    i. Probe: How confident do you feel counseling pregnant farmworker patients on occupational pesticide exposure?
      ii. Probe: Do you feel like it is your responsibility to screen and counsel pregnant farmworking patients for occupational pesticide exposure?  
      1. If you don’t think it is your responsibility, who do you think it should be?
      iii. Probe: How do you currently counsel your pregnant farmworking patients? (eg, verbal, handouts, videos)
      1.   Follow up: What do you think would be the ideal way to counsel and edutcate your patients?
  - Content #2- Provider support in reducing pesticide exposure: Have you supported pregnant farmworkers in reducing occupational pesticide exposure?  
    i. Probe: Have you written a work accommodation note?
      ii. Probe: Are you familiar with the eligibility criteria for state disability insurance for occupational pesticide exposure during pregnancy?
      iii. Probe: Have you certified a state disability insurance (SDI) claim?
      1.   Probe: What was your experience with that?
      2.  Probe: At what gestational age do you certify SDI claims?
      3.  Probe: What problems or barriers did you encounter?
    iv. Probe: For those that do no certify SDI claims, can you share your thinking about that?
  - Content #3- Improvement: How can we improve the process of protecting pregnant farmworkers from pesticide exposure? 
    i. Probe: How can SDI certification/ interfacing with the Employment Development Department be improved?
      ii. Probe: What kinds of system changes in your own clinic/work environment would you suggest?
      iii. Probe: What policy changes might support these improvements? (eg, health plan payers, state policies, Medi-Cal)
      iv. Probe: What kind of continuing medical education would help you and your staff support these improvements?
  - Content #4- Knowledge of resources: What resources do you use to inform your clinical practice in caring for pregnant farmworkers, specifically related to pesticide exposure? 
    i. Probe: Please tell me more.
      ii. Probe: What resources would enable you to provide better care?
4. Closing/debriefing: What else would you like to tell me about?

### 2: Interview Guide

1. Please share your first name, professional title or role, and 1 thing you love about your profession?
2. Content questions
  - What is your involvement in helping pregnant farmworkers to mitigate their occupational pesticide exposure?
  - If they are involved in healthcare: 
    i. Content #1- Comfort and responsibility on counseling for pesticide exposure: Do you screen and counsel your pregnant farmworker patients on the health effects of pesticide expo sure?    
      1. Probe: How confident do you feel counseling pregnant farmworker patients on occupational pesticide exposure?
      2.  Probe: Do you feel like it is your responsibility to screen and counsel pregnant farmworking patients for occupational pesticide exposure?  
        - If you don’t think it is your responsibility, who do you think it should be?
      3.  Probe: How do you currently counsel your pregnant farmworking patients? (eg, verbal, handouts, videos)  
        - Follow up: What do you think would be the ideal way to counsel and educate your patients?
    ii. Content #2- Provider support in reducing pesticide exposure: Have you supported pregnant farmworkers in reducing occupational pesticide exposure?  
      1. Probe: Have you written a work accommodation note?
      2.  Probe: Are you familiar with the eligibility criteria for state disability insurance for occupational pesticide exposure during pregnancy?
      3.  Probe: Have you certified a state disability insurance (SDI) claim?
        - Probe: What was your experience with that?
        - Probe: At what gestational age do you certify SDI claims?
        - Probe: What problems or barriers did you encounter?
      4.  Probe: For those that do no certify SDI claims, can you share your thinking about that?
    iii. Content #3- Improvement: How can we improve the process of protecting pregnant farmworkers from pesticide exposure?   
      1. Probe: How can SDI certification/ interfacing with the Employment Development Department be improved?
      2.  Probe: What kinds of system changes in your own clinic/work environment would you suggest?
      3.  Probe: What policy changes might support these improvements? (eg, health plan payers, state policies, Medi-Cal)
      4.  Probe: What kind of continuing medical education would help you and your staff support these improvements?
     iv. Content #4- Knowledge of resources: What resources do you use to inform your clinical practice in caring for pregnant farmworkers, specifically related to pesticide exposure?   
      1. Probe: Please tell me more.
      2.  Probe: What resources would enable you to provide better care?
  - If they are involved in SDI certification processes:
     i. Can you tell me about your involve ment in the SDI certification process?
     ii. What are facilitators to approving pesticide-related SDI claims for pregnant farmworkers?
     iii. What are barriers to approving pesticide-related SDI claims for pregnant farmworkers?
     iv. How can the process of SDI certification be improved?
  - If they are a member of a farmworker community organization:
     i. Please tell me about your work.
    ii. What are barriers people in your community face that inhibits them from reducing their occupational pesticide exposure during pregnancy?
     iii. How can we better support pregnant farmworkers?
3. Closing/debriefing: What else would you like to tell me about?

## References

[1] Centers for Disease Control and Prevention (CDC), National Institute for Occupational Safety and Health (NIOSH). Pesticides - reproductive health. 2023. Accessed November 9, 2023. https://www.cdc.gov/niosh/topics/repro/pesticides.html

[2] Bouchard MF Chevrier J Harley KG , et al. Prenatal exposure to organophosphate pesticides and IQ in 7-year-old children. Environ Health Perspect. 2011;119(8):1189-1195. doi:10.1289/ehp.1003185 21507776 PMC3237357

[3] Sagiv SK Kogut K Harley K Bradman A Morga N Eskenazi B . Gestational exposure to organophosphate pesticides and longitudinally assessed behaviors related to attention-deficit/hyperactivity disorder and executive function. Am J Epidemiol. 2021;190(11):2420-2431. doi:10.1093/aje/kwab173 34100072 PMC8757311

[4] Marks AR Harley K Bradman A , et al. Organophosphate pesticide exposure and attention in young Mexican-American children: the CHAMACOS study. Environ Health Perspect. 2010;118(12):1768-1774. doi:10.1289/ehp.1002056 21126939 PMC3002198

[5] Albadrani MS Aljassim MT El-Tokhy AI . Pesticide exposure and spontaneous abortion risk: a comprehensive systematic review and meta-analysis. Ecotoxicol Environ Saf. 2024;284:117000. doi:10.1016/j.ecoenv.2024.117000 39265264

[6] Simões M Vermeulen R Portengen L Janssen N Huss A . Exploring associations between residential exposure to pesticides and birth outcomes using the Dutch birth registry. Environ Int. 2023;178:108085. doi:10.1016/j.envint.2023.108085 37421898

[7] Sagiv SK Mora AM Rauch S , et al. Prenatal and childhood exposure to organophosphate pesticides and behavior problems in adolescents and young adults in the CHAMACOS study. Environ Health Perspect. 2023;131(6):067008. doi:10.1289/EHP11380 PMC10259762 37307167

[8] Coker E Gunier R Bradman A , et al. Association between pesticide profiles used on agricultural fields near maternal residences during pregnancy and IQ at age 7 years. Int J Environ Res Public Health. 2017;14(5):506. doi:10.3390/ijerph14050506 28486423 PMC5451957

[9] Engel SM Wetmur J Chen J , et al. Prenatal exposure to organophosphates, paraoxonase 1, and cognitive development in childhood. Environ Health Perspect. 2011;119(8):1182-1188. doi:10.1289/ehp.1003183 21507778 PMC3237356

[10] Gunier RB Bradman A Harley KG Kogut K Eskenazi B . Prenatal residential proximity to agricultural pesticide use and IQ in 7-year-old children. Environ Health Perspect. 2017;125(5):057002. doi:10.1289/EHP504 PMC5644974 28557711

[11] Rauh V Arunajadai S Horton M , et al. Seven-year neurodevelopmental scores and prenatal exposure to chlorpyrifos, a common agricultural pesticide. Environ Health Perspect. 2011;119(8):1196-1201. doi:10.1289/ehp.1003160 21507777 PMC3237355

[12] Gerona RR Reiter JL Zakharevich I , et al. Glyphosate exposure in early pregnancy and reduced fetal growth: a prospective observational study of high-risk pregnancies. Environ Health. 2022;21(1):95. doi:10.1186/s12940-022-00906-3 36221133 PMC9552485

[13] Stillerman KP Mattison DR Giudice LC Woodruff TJ . Environmental exposures and adverse pregnancy outcomes: a review of the science. Reprod Sci. 2008;15(7):631-650. doi:10.1177/1933719108322436 18836129

[14] Arcury TA Laurienti PJ Chen H , et al. Organophosphate pesticide urinary metabolites among Latino immigrants: North Carolina farmworkers and non-farmworkers compared. J Occup Environ Med. 2016;58(11):1079-1086. doi:10.1097/JOM.0000000000000875 27820757 PMC5113293

[15] Arcury TA Laurienti PJ Talton JW , et al. Pesticide urinary metabolites among Latina farmworkers and nonfarmworkers in North Carolina. J Occup Environ Med. 2018;60(1):e63-e71. doi:10.1097/JOM.0000000000001189 PMC5758422 29023343

[16] Hoppin JA Adgate JL Eberhart M Nishioka M Ryan PB . Environmental exposure assessment of pesticides in farmworker homes. Environ Health Perspect. 2006;114(6):929-935. doi:10.1289/ehp.8530 16759997 PMC1480520

[17] California Department of Food and Agriculture. Statistics. 2025. Accessed September 1, 2025. https://www.cdfa.ca.gov/Statistics/

[18] Ornelas I Fung W Gabbard S Carroll D . California findings from the National Agricultural Workers Survey (NAWS) 2015–2019: a demographic and employment profile of california farmworkers. Research report no. 15. 2022. Accessed September 3, 2025. https://www.dol.gov/sites/dolgov/files/ETA/publications/ETAOP2022-15_NAWS_Research_Report_15_508c.pdf

[19] Ferguson R Dahl K DeLonge M . Farmworkers at risk: the growing dangers of pesticides and heat. Union of Concerned Scientists. 2019. Accessed October 15, 2025. https://www.ucs.org/resources/farmworkers-at-risk

[20] National Farm Worker Ministry. Farm worker conditions in California. Published April 17, 2009. Accessed August 25, 2025. https://nfwm.org/news/farm-worker-conditions-in-california/

[21] Masterson AR Lopez S Garcia G Maldonado A . Maternal health rights for farmworker women: farmworker voices from the field. evaluation report. University of California Global Health Institute. 2023. Accessed October 15, 2025. https://ucghi.universityofcalifornia.edu/pdf/farmworker_maternal_health_and_rights_evaluation_report_november_2023.pdf

[22] California Civil Rights Department. Reasonable accommodation. 2025. Accessed August 27, 2025. https://calcivilrights.ca.gov/accommodation/

[23] U.S. Equal Employment Opportunity Commission (EEOC). Pregnancy discrimination and pregnancy-related disability discrimination. 2025. Accessed November 15, 2023. https://www.eeoc.gov/pregnancy-discrimination

[24] California Employment Development Department (EDD). Disability insurance – eligibility FAQs. 2025. Accessed August 25, 2025. https://edd.ca.gov/en/disability/FAQ_DI_Eligibility/

[25] California Employment Development Department (EDD). Disability insurance benefit payment amounts. 2025. Accessed September 6, 2025. https://edd.ca.gov/en/disability/Calculating_DI_Benefit_Payment_Amounts/

[26] Stotland NE Sutton P Trowbridge J , et al. Counseling patients on preventing prenatal environmental exposures–a mixed-methods study of obstetricians. PLoS One. 2014;9(6):e98771. doi:10.1371/journal.pone.0098771 PMC4070906 24964083

[27] Grindler NM Allshouse AA Jungheim E Powell TL Jansson T Polotsky AJ . OBGYN screening for environmental exposures: a call for action. PLoS One. 2018;13(5):e0195375. doi:10.1371/journal.pone.0195375 PMC5955561 29768418

[28] American College of Obstetricians and Gynecologists’ Committee on Obstetric Practice. Reducing prenatal exposure to toxic environmental agents: ACOG committee opinion, number 832. Obstet Gynecol. 2021;138:e40-e54. 10.1097/AOG.0000000000004449 34259492

[29] American College of Obstetricians and Gynecologists (ACOG). Committee Opinion No. ACOG committee opinion no. 733: employment considerations during pregnancy and the postpartum period. Obstet Gynecol. 2018;131:e115-e123. 10.1097/AOG.0000000000002589 29578986

[30] Sanborn M Grierson L Upshur R , et al. Family medicine residents’ knowledge of, attitudes toward, and clinical practices related to environmental health: multi-program survey. Can Fam Physician. 2019;65(6):e269-e277. PMC6738382 31189641

[31] Chambers JY Rippon J Ahle D Le X Miller B Moreno A . Is the future green? assessing environmental health confidence in internal medicine residents. J Grad Med Educ. 2024;16(6s):99-103. doi:10.4300/JGME-D-24-00081.1 39677903 PMC11644584

[32] Center for WorkLife Law. Guidance for medical care providers: supporting farmworkers during pregnancy and postpartum – pregnant@work. 2020. Accessed November 9, 2023. https://pregnantatwork.org/guidance-medical-provider-agriculture/

[33] California Office of Environmental Health Hazard Assessment (OEHHA). Essential information for healthcare providers – pregnant agricultural workers and pesticides. 2025. Accessed November 9, 2023. https://oehha.ca.gov/sites/default/files/media/downloads/pesticides/general-info/pregnancypesticidesinfosheet.pdf

[34] Tong A Sainsbury P Craig J . Consolidated criteria for reporting qualitative research (COREQ): a 32-item checklist for interviews and focus groups. Int J Qual Health Care. 2007;19(6):349-357. doi:10.1093/intqhc/mzm042 17872937

[35] Maxwell JA , ed. Qualitative Research Design: An Interactive Approach. 3rd ed. Sage Publications; 2013.

[36] Corbin J Strauss A , eds. Basics of Qualitative Research: Techniques and Procedures for Developing Grounded Theory. 3rd ed. Sage Publications; 2008.

[37] Creswell JW Poth CN , eds. Qualitative Inquiry and Research Design: Choosing Among Five Approaches. 5th ed. Sage Publications; 2018.

[38] Lee J Donlan W Cardoso EE Paz JJ . Cultural and social determinants of health among indigenous Mexican migrants in the United States. Soc Work Public Health. 2013;28(6):607-618. doi:10.1080/19371918.2011.619457 23944171

[39] Maxwell AE Young S Moe E Bastani R Wentzell E . Understanding factors that influence health care utilization among Mixtec and Zapotec women in a farmworker community in California. J Community Health. 2018;43(2):356-365. doi:10.1007/s10900-017-0430-8 28975501 PMC5832539

[40] California Department of Managed Health Care. Language assistance. 2025. Accessed July 27, 2025. https://www.dmhc.ca.gov/HealthCareinCalifornia/YourHealthCareRights/LanguageAssistance.aspx

[41] Fung W Prado K Gold A Padovani A Carroll D Finchum-Mason E . Findings from the national agricultural workers survey (NAWS) 2021–2022: a demographic and employment profile of united states crop workers. Research report no. 17. U.S. Department of Labor, Employment and Training Administration. 2023. Accessed October 15, 2025. https://www.dol.gov/sites/dolgov/files/ETA/naws/pdfs/NAWS%20Research%20Report%2017.pdf

[42] Cha P . Health care access among California’s farmworkers. Policy Brief. Public Policy Institute of California; 2022. Accessed October 15, 2025. https://www.ppic.org/publication/health-care-access-among-californias-farmworkers/

[43] University of California, Merced, Community & Labor Center. Farmworker health in California: health in a time of contagion, drought, and climate change. Published 2022. Accessed October 15, 2025. https://clc.ucmerced.edu/sites/g/files/ufvvjh626/f/page/documents/fwhs_report_2.2.2383.pdf

[44] Nwadiuko J German J Chapla K , et al. Changes in health care use among undocumented patients, 2014-2018. JAMA Netw Open. 2021;4(3):e210763. doi:10.1001/jamanetworkopen.2021.0763 PMC7936260 33666662

[45] Kelley MA Flocks JD Economos J McCauley LA . Female farmworkers’ health during pregnancy: health care providers’ perspectives. Workplace Health Saf. 2013;61(7):308-313. doi:10.1177/216507991306100706 23799657

[46] California Office of Environmental Health Hazard Assessment (OEHHA). Health education resources. 2021. Accessed August 19, 2025. https://oehha.ca.gov/pesticides/health-education-resources

