# Supplementary material for: Examining Healthcare Providers’ Knowledge, Attitudes, and Practices in Supporting Pregnant Farmworkers to Mitigate Occupational Pesticide Exposure in California: A Qualitative Study
Source: J Prim Care Community Health. 2025 Dec 23;16:21501319251407545. doi: 10.1177/21501319251407545 (PMC12744005; doi:10.1177/21501319251407545)
Supplement: sj-docx-1-jpc-10.1177_21501319251407545 – Supplemental material for Examining Healthcare Providers’ Knowledge, Attitudes, and Practices in Supporting Pregnant Farmworkers to Mitigate Occupational Pesticide Exposure in California: A Qualitative Study [file sj-docx-1-jpc-10.1177_21501319251407545.docx]

Supplemental Material for “Examining healthcare providers’ knowledge, attitudes, and practices in supporting pregnant farmworkers to mitigate occupational pesticide exposure in California: a qualitative study”

Supplemental Table 1: Access to Care Barriers Supporting Quotes

| Theme | Supporting Quotes |
| --- | --- |
| Language barriers | “[A] Spanish speaking, or Mixteco speaking [patient], is probably not going to be like, ‘Oh, is there any information that you can give me?’ From my experience, they don't ask those types of questions. So just another lack of help that they are not receiving, basically.”- P5 |
| Lack of interpreters | “There's so many different [languages] that sometimes we don't get it. There's no one available to translate for that language.”-P16  “I've had families that have traveled really far distances to come to our hospital, so that probably affects our numbers as well, but because we have the Mixteco speaking translators, patients and families come pretty far to come get services with us.”-P6 |
| Transportation issues | “I think a lot also has to do with their access to care, you know, and just having car rides to get to their appointments.”-P1 |
| Financial considerations | “But again, you know, being the kid of farm workers, and actually, having spent some summers doing it myself, and just from speaking with patients, I just don't see them [patients] missing work. Because it just seems like the name of the game is working as much as you can for as long as you can, so that you can earn that money. I think that honestly, that whatever health concerns they have just aren't really thought about or addressed because the whole purpose of them being here is working, working, working, working. The harvest time is so short that they just want to make sure that they get as much as they can during that time.”- P10 |

Supplemental Table 2: Facilitators to Prenatal Occupational Pesticide Exposure Screening Supporting Quotes

| Theme | Supporting Quotes |
| --- | --- |
| Staff support | “...our CPSP, who does the initial intake…and so, when the patient comes to see me for example, the chart is basically ready with her previous medical history or prenatal or OB history. And so during that time, usually by time I review the chart and see the patient I have an idea what occupation she is involved in. And so when I go in and I do my initial assessment, that's basically when I briefly talk to her about it.”-P2  “We have CPSP visits that are dedicated for our patients. When they come in, they'll usually do it and all their information will be in the chart, which is really helpful. And then there's dedicated appointments that they have moving forward.”-P11  “Most of the initial encounters about assessment, risk assessment and prenatal screening are our CPSP.”-P15 |
| Screening integrated into clinical workflows | “I usually screen just for occupational exposure at that first prenatal visit. Do you work? If so, what kind of work? How is that going to impact your pregnancy? And how is pregnancy going to impact your ability to work?”-P8  “I do screen, but not in the sense of thinking specifically for farm workers, but I think about their occupation in general”-P11  “We ask if the patient is a field worker. We ask for the general background.”-P15 |

Supplemental Table 3: Barriers to Prenatal Occupational Pesticide Exposure Screening Supporting Quotes

| Theme | Supporting Quotes |
| --- | --- |
| Visit time constraints | “...another is because of the lack of time. Our schedule, most of the time, is overbooked, and we don't have the time. I could say we can find the time, but most of the time we are super booked, and we have very limited time to assess our patients.”-P15 |
| Provider lack of training and knowledge | “I was just about to ask what her [other participant] recommendations were and what the screening questions were, because I actually don't have any questions that I ask them”-P4 |
| Improper setting | “…in the hospital setting, and we do not screen our patients for their pesticide exposure at all…because we're seeing them for the most part at the end of their pregnancy.”-P3  “Being a labor and delivery nurse, I don't do any screening at all”-P14  “I would say that I do not routinely talk about that. And I think my role is a little bit unique just because I'm not the primary provider for these patients.”-P1 |

Supplemental Table 4: Occupational Pesticide Exposure Counseling Supporting Quotes

| Theme | Supporting Quotes |
| --- | --- |
| Shared decision making | “...trying to assess what their exposure is and then determining if there is there an alternate part of the work they could do, or is there a way they could, you know, make sure they're not out when the grapes are getting sprayed. Or washing their hands before they eat, like things like that, to really reduce that risk.”- P8  “They talk about what exactly they're doing in the field and what exposures they might have and what accommodations they might need. And I usually write, need access to bathroom, clean water , hand washing, masks, and gloves in an early letter. And then talk about disability whenever they feel like they need it, or if they can't meet those accommodations.”-P8  “Ever since we started working on this and the pesticide exposure, and then the switch in SDI, I think there's been a push to share with the patients with shared decision making to decide if they want to go out of work earlier.”-P9 |
| Patient-provider power dynamics | “And I would say in this patient population, I don't get any pushback on it.”-P1 |

Supplemental Table 5: Barriers to Certifying State Disability Insurance Claims Supporting Quotes

| Theme | Supporting Quotes |
| --- | --- |
| Lack of time in clinic to fill out paperwork | “Doctors have so many patients to see that it's really hard for them to stop and pull all the documentation up and do it and redo paperwork”- P9  “It takes time to fill it out, it's hard to find a time during the day where you can actually sit down and do that form. We see about 28 to 30 patients.”-P17 |
| Lack of provider knowledge and training | “I think most people don't even know that they can do it [certify SDI claims]. I think a lot of people miss out on that opportunity.”-P9  “I think there's a lot of hesitation on the OB provider side that they are doing something fraudulent or not doing what's right, you know, working the system or something like that.”-P1 |
| Financial stressors on the patient | “They've already stopped working, and then here they're going months without pay, and it's just super stressful for families.”-P8  “You know, a lot of them are sending home money to their families. So even a small reduction is a big deal.”-P9  “I know a lot of our migrant workers don't want to stop working because of the fact that they won't get any money back, or they'll get very minimal benefits.”-P7  “I have patients that have submitted claims like, let's say, in November, and they still haven't received a response. And since that's their only income, they’re suffering financial loss.”-P16  “You don't qualify [for SDI] if you're working, so you can't really just see if you're eligible. You have to stop working and then find out on the back end if they're going to give it [disability] to you. I think that's a risk that you have to take at the front end.”-P9 |
